# Association of depressive disorder with biochemical and anthropometric indices in adult men and women

**DOI:** 10.1038/s41598-021-93103-0

**Published:** 2021-06-30

**Authors:** Bum Ju Lee

**Affiliations:** Future Medicine Division, Korea Institute of Oriental Medicine, 1672 Yuseong-daero, Yuseong-gu, Daejeon, 34054 Republic of Korea

**Keywords:** Diseases, Health care, Medical research, Risk factors

## Abstract

Depression is a common psychiatric disorder. Although many risk factors for depression have been reported, the associations of biochemical and anthropometric indices with depressive disorder remain unclear. The objective of this study was to assess whether there are significant associations of depressive disorder with biochemical and obesity indices. This study was based on data from the Korea National Health and Nutrition Examination Survey from 2007 to 2018, and logistic regression was performed to examine the association of depression with biochemical and obesity indices. A total of 33,993 subjects were included in the analyses. Study subjects consisted of 13,178 men in the control group (mean age of 51.12 years), 509 men in the depression group (53.67), 18,279 women in the control group (50.5), and 2027 women in the depression group (55.39). Among men, the depression group was significantly more likely to have a lower height and weight than the control group. Compared to the control group, the depression group was more likely to have higher triglyceride levels and tended to have lower hematocrit and blood urea nitrogen (BUN) levels. Among women, the depression group was more likely to have higher triglyceride, aspartate aminotransferase (AST), BUN, and creatinine levels and lower high-density lipoprotein cholesterol (HDL-C), hematocrit, and red blood cell counts. Several biochemical and anthropometric indices used in this study were associated with depressive disorder, but these associations may differ according to sex.

## Introduction

Depressive disorder is a leading cause of disease burden worldwide^1–4^. Depression is estimated to affect 322–350 million people worldwide^2,3^, and the disorder is projected to be the greatest contributor to disease burden by 2030^3,5^. Globally, depressive disorder is one of the most common psychiatric disorders and is associated with feelings of guilt, depressive status, increased fatigability, anxiety, loss of interest, and poor self-worth^2^. Depressive disorder is associated with various conditions, such as suicide^2,4^, obesity^6^, hypertension and stroke^1,7^, cardiovascular diseases^8,9^, ischemic heart disease^1,4^, myocardial infarction^10^, Alzheimer’s disease^11^, and Parkinson’s disease^12^, and all-cause mortality in elderly men^13^.

Generally, known risk factors for depression consist of 4 categories. The first category covers socioeconomic risk factors such as sex (more common among women), low income, low education level, noneconomic activity, divorce and bereavement, smoking and nicotine-dependence symptoms, alcohol consumption, ethnic group, and occupation level^2,3,5,14–19^. The second includes anthropometric factors related to obesity, such as body mass index (BMI), waist circumference, and weight^2,3,6,20–28^. The third category covers biochemical factors such as platelets, total cholesterol, triglycerides, high-density lipoprotein cholesterol (HDL-C), red blood cells (RBCs), white blood cells (WBCs), hemoglobin, hematocrit, glucose, alanine transaminase (ALT), and blood urea nitrogen (BUN)^29–38^. The final category includes genetic factors^2,39^. For example, the risk of depression increases after divorce and/or bereavement^14^. Major risk factors for depression are female sex, alcohol consumption, smoking, poverty, Caucasian race, and chronic disease^15,17^.

However, although numerous previous studies have examined the associations of obesity and biochemical indices with depressive disorder, the associations remain debatable. The available literature reports different patterns of association between anthropometric or obesity indices and depressive disorder: no significant associations^29,31,40–42^ and significant associations^6,21–28^ have been reported. Additionally, the links between depression and biochemical indices are very confusing and controversial. For example, triglyceride levels were closely linked to depressive disorder^31,32,35^ but not to depression^33,41^. Additionally, HDL-C levels were related to the disorder^33–36^ but were also found not to be associated^31,41^. RBCs were associated with depression^29,37,38^ but were also found not to be associated^41^. Therefore, the objective of this study was to assess whether there are significant associations of depression disorder with biochemical and anthropometric indices in a large-scale cross-sectional study. Our findings may contribute to a better understanding of depressive disorder risks in the Korean population.

## Results

The overall sociodemographic characteristics of the study sample are described in Table 1. A total of 33,993 subjects were included in the final analyses. The study subjects consisted of 13,178 men in the control group, 509 men in the depression group, 18,279 women in the control group, and 2027 women in the depression group. The mean (standard deviation, SD) ages of the control and depression groups in this study were 51.12 (16.29) and 53.67 (16.62) years in men and 50.5 (15.95) and 55.39 (14.74) years in women. Income, education, occupation, economic activity, stress, and alcohol consumption were significantly associated with depressive disorder in men, and all sociodemographic variables used in this study were associated with the disease in women.

**Table 1:** Sociodemographic characteristics of the study sample.

Variable	Men			Women		
	Control	Depression		Control	Depression	
Subjects (n)	13,178	509		18,279	2027	
Age (y)	51.12 (16.29)	53.67 (16.62)		50.5 (15.95)	55.39 (14.74)	
Body mass index (kg/m^2^)	24.37 (3.261)	24.39 (3.459)		23.48 (3.57)	23.97 (3.519)	
Number of household members (n)	2.969 (1.187)	2.802 (1.29)		3 (1.244)	2.76 (1.269)	
Systolic BP (mmHg)	121 (15.24)	121.1 (15.06)		116.6 (17.77)	119.3 (17.7)	
Diastolic BP (mmHg)	77.68 (10.44)	77.73 (9.731)		73.57 (9.782)	74.51 (9.842)	
Height (cm)	170.1 (6.779)	168.4 (6.81)		157.1 (6.484)	155.8 (6.283)	
Weight (kg)	70.7 (11.51)	69.4 (12.17)		57.95 (9.237)	58.19 (9.445)	
Waist circumference (cm)	85.84 (8.917)	86.17 (9.76)		78.91 (9.801)	80.7 (9.869)	
Glucose (mg/dl)	103.4 (24.97)	101.8 (24.03)		97.79 (21.45)	99.04 (22.16)	
Total cholesterol (mg/dl)	188.8 (36.8)	191.3 (38.89)		192.2 (36.62)	193 (37.8)	
High-density lipoprotein cholesterol (HDL-C) (mg/dl)	47.18 (11.27)	46.67 (12.02)		53.72 (12.48)	51.45 (12.87)	
Triglycerides (mg/dl)	158.9 (129.1)	173.7 (144.1)		115.5 (79.9)	130.9 (98.04)	
Aspartate aminotransferase (AST) (IU/l)	25.02 (16.18)	25.96 (14.61)		20.98 (9.708)	22.49 (17.76)	
Alanine aminotransferase (ALT) (IU/l)	26.74 (21.65)	27.46 (19.74)		18.01 (13.19)	19.55 (14.13)	
Hemoglobin (g/dl)	15.22 (1.263)	15.08 (1.281)		13.1 (1.147)	13.12 (1.103)	
Hematocrit (%)	45.47 (3.613)	44.81 (3.736)		39.92 (3.132)	39.66 (3.042)	
Blood urea nitrogen (BUN) (mg/dl)	15.38 (4.73)	14.88 (4.419)		13.99 (4.54)	14.3 (4.497)	
Creatinine (mg/dl)	0.972 (0.325)	0.97 (0.179)		0.717 (0.212)	0.743 (0.229)	
White blood cell (WBC) (Thous/ul)	6.609 (1.821)	6.68 (1.739)		5.887 (1.64)	5.946 (1.8)	
Red blood cells (RBC) (Mil/ul)	4.905 (0.453)	4.832 (0.455)		4.357 (0.346)	4.318 (0.353)	
**Income (quintile)**			< 0.001			< 0.001
Low	2564 (18.7)	163 (1.2)		3502 (17.2)	512 (2.5)	
Lower-middle	2610 (19.1)	97 (0.7)		3691 (18.2)	392 (1.9)	
Middle	2633 (19.2)	76 (0.6)		3782 (18.6)	369 (1.8)	
Upper-middle	2691 (19.7)	76 (0.6)		3678 (18.1)	392 (1.9)	
High	2680 (19.6)	97 (0.7)		3626 (17.9)	362 (1.8)	
**Education**			< 0.001			< 0.001
Elementary school or less	2068 (15.1)	130 (0.9)		4806 (23.7)	830 (4.1)	
Middle school	1427 (10.4)	78 (0.6)		1879 (9.3)	287 (1.4)	
High school	4547 (33.2)	169 (1.2)		5689 (28)	591 (2.9)	
University or higher	5136 (37.5)	132 (1)		5905 (29.1)	319 (1.6)	
**Occupation**			< 0.001			< 0.001
Managers and professionals	2167 (15.8)	55 (0.4)		2174 (10.7)	101 (0.5)	
Clerks	1612 (11.8)	21 (0.2)		1527 (7.5)	83 (0.4)	
Service workers and sale workers	1343 (9.8)	41 (0.3)		2764 (13.6)	206 (1.0)	
Skilled agricultural and fishery workers	897 (6.6)	45 (0.3)		718 (3.5)	101 (0.5)	
Craft and machine operators	2622 (19.2)	66 (0.5)		510 (2.5)	48 (0.2)	
Elementary occupations	1043 (7.6)	36 (0.3)		1711 (8.4)	226 (1.1)	
Unemployed	3494 (25.5)	245 (1.8)		8875 (43.7)	1262 (6.2)	
**Marital status**			0.193			< 0.001
Married	10,735 (78.4)	403 (2.9)		16,027 (78.9)	1894 (9.3)	
Single	2443 (17.8)	106 (0.8)		2250 (11.1)	133 (0.7)	
No response				2 (0)	0 (0)	
**Economic activity**			< 0.001			< 0.001
Yes	9684 (70.8)	264 (1.9)		9404 (46.3)	765 (3.8)	
No (unemployed)	3494 (25.5)	245 (1.8)		8875 (43.7)	1262 (6.2)	
**Stress**			< 0.001			< 0.001
Low	10,023 (73.2)	278 (2)		12,949 (63.8)	1045 (5.1)	
High	3155 (23.1)	231 (1.7)		5330 (26.2)	982 (4.8)	
**Smoking**			0.075			< 0.001
Nonsmoking	8431 (61.6)	306 (2.2)		17,392 (85.6)	1858 (9.2)	
Smoking	4747 (34.7)	203 (1.5)		887 (4.4)	169 (0.8)	
**Alcohol consumption**			< 0.001			
Less than once a month	3724 (27.2)	202 (1.5)		10,797 (53.2)	1355 (6.7)	< 0.001
More than once a month	9454 (69.1)	307 (2.2)		7482 (36.8)	672 (3.3)	
**Diabetes diagnosis**			0.535			< 0.001
No	11,815 (86.3)	452 (3.3)		16,881 (83.1)	1806 (8.9)	
Yes	1363 (10)	57 (0.4)		1398 (6.9)	221 (1.1)	
**Hypertension diagnosis**			0.233			
No	9912 (72.4)	371 (2.7)		14,335 (70.6)	1439 (7.1)	< 0.001
Yes	3266 (23.9)	138 (1)		3944 (19.4)	588 (2.9)	
**Menstruation**						< 0.001
No				17,066 (84)	1938 (9.5)	
Yes				1213 (6)	89 (0.4)	
**Pregnancy**						0.006
No				18,177 (89.5)	2025 (10)	
Yes				102 (0.5)	2 (0)	

Continuous and categorical variables are expressed as the mean (standard deviation) and frequency (percentage).

p-values were obtained from the chi-square tests for categorical variables.

Table 2 describes the association of depressive disorder with anthropometric and biochemical indices in men. The depressive disorder group was significantly older and tended to have a lower number of household members than the control group. Regarding anthropometric indices, compared to the control group, the depressive disorder group was significantly more likely to have a lower height and weight in the crude model. These associations maintained their significance in model 1, which was adjusted for age and BMI, and model 2, which was adjusted for age, BMI, income, education, occupation, number of household members, marital status, stress, smoking, and alcohol consumption. However, BMI and waist circumference were not associated with depression.

**Table 2:** Association of depression disorder with biochemical and anthropometric indices in men.

Variable	Crude model		Model 1		Model 2	
	OR (95% CI)	p	Adj. OR (95% CI)	Adj. p	Adj. OR (95% CI)	Adj. p
Age	1.17 (1.07–1.28)	0.001				
Body mass index	1.01 (0.92–1.10)	0.885				
Number of household members	0.87 (0.79–0.95)	0.002	0.90 (0.82–0.99)	0.030		
Systolic BP	1.01 (0.92–1.10)	0.873	0.95 (0.87–1.05)	0.324	0.95 (0.86–1.04)	0.249
Diastolic BP	1.01 (0.92–1.10)	0.912	1.03 (0.94–1.13)	0.541	1.11 (1.01–1.22)	0.034
Height	0.78 (0.72–0.85)	< 0.001	0.79 (0.72–0.88)	< 0.001	0.87 (0.79–0.97)	0.010
Weight	0.89 (0.81–0.98)	0.013	0.64 (0.52–0.79)	< 0.001	0.77 (0.62–0.95)	0.014
Waist circumference	1.04 (0.95–1.13)	0.412	1.02 (0.85–1.23)	0.834	0.98 (0.82–1.19)	0.864
Glucose	0.93 (0.84–1.03)	0.149	0.88 (0.78–0.98)	0.019	0.88 (0.79–0.98)	0.017
Total cholesterol	1.07 (0.98–1.17)	0.130	1.09 (1.00–1.19)	0.065	1.13 (1.04–1.24)	0.005
HDL-C	0.96 (0.87–1.05)	0.317	0.97 (0.88–1.06)	0.521	1.03 (0.93–1.13)	0.615
Triglycerides	1.10 (1.02–1.18)	0.012	1.11 (1.03–1.19)	0.008	1.12 (1.03–1.20)	0.005
AST	1.04 (0.98–1.10)	0.208	1.04 (0.97–1.10)	0.269	1.02 (0.95–1.10)	0.555
ALT	1.03 (0.95–1.11)	0.459	1.05 (0.97–1.13)	0.230	1.02 (0.94–1.10)	0.645
Hemoglobin	0.90 (0.83–0.98)	0.020	0.94 (0.86–1.04)	0.218	0.98 (0.90–1.08)	0.714
Hematocrit	0.84 (0.77–0.91)	< 0.001	0.87 (0.79–0.95)	0.002	0.90 (0.82–0.99)	0.028
BUN	0.88 (0.80–0.98)	0.016	0.82 (0.74–0.91)	< 0.001	0.87 (0.79–0.96)	0.008
Creatinine	0.99 (0.90–1.09)	0.895	0.98 (0.88–1.09)	0.713	0.97 (0.87–1.08)	0.583
WBC	1.04 (0.95–1.13)	0.388	1.05 (0.96–1.14)	0.310	0.96 (0.88–1.05)	0.370
RBC	0.85 (0.78–0.93)	< 0.001	0.89 (0.80–0.98)	0.015	0.91 (0.83–1.01)	0.073

*HDL-C* high-density lipoprotein cholesterol, *AST* aspartate aminotransferase, *ALT* alanine aminotransferase, *BUN* blood urea nitrogen, *WBC* white blood cell, *RBC*: red blood cell.

Crude Model: unadjusted model, Model 1: adjusted for age and BMI, Model 2: adjusted for age, BMI, income, education, occupation, number of household members, marital status, stress, smoking, and alcohol consumption.

Among the biochemical indices, glucose was not associated with depression in the crude model, but glucose was significantly related to depression in models 1 and 2. The total cholesterol level was not related to depression in the crude model or model 1, but an association between total cholesterol level and depression was observed in model 2. The depression group was significantly more likely to have higher triglyceride levels than the control group in all models. The hematocrit level was lower in the depression group than in the control group in all models. The hemoglobin level was associated with depression in the crude model, but this association disappeared in models 1 and 2. Additionally, the depression group was more likely to have lower BUN levels than the control group in all models. The RBC count was lower in the depression group than in the control group in the crude model, but this association was attenuated in model 1 and became nonsignificant in model 2.

Table 3 shows an association of depression disorder with anthropometric and biochemical indices in women. Model 2 in women was adjusted for all confounders in model 2 in men plus menstruation and pregnancy. Compared to the control group, the depressive disorder group was older and had a lower number of household members. Height and weight in women were not associated with depression disorder, which was different from the findings in men. Although the depression group tended to have a lower height than the control group in the crude model, this association was nonsignificant in models 1 and 2. Additionally, waist circumference and systolic blood pressure (BP) were associated with depression, but these associations disappeared in models 1 and 2. Diastolic BP was associated with depression in the crude model, but the significant association was attenuated in model 1 and became nonsignificant in model 2.

**Table 3:** Association of depression disorder with biochemical and anthropometric indices in women.

Variable	Crude model		Model 1		Model 2	
	OR (95% CI)	p	Adj. OR (95% CI)	Adj. p	Adj. OR (95% CI)	Adj. p
Age	1.37 (1.31–1.44)	< 0.001				
Body mass index	1.14 (1.09–1.19)	< 0.001				
Number of household members	0.82 (0.78–0.86)	< 0.001	0.93 (0.88–0.98)	0.004		
Systolic BP	1.16 (1.11–1.21)	< 0.001	0.97 (0.92–1.03)	0.338	0.95 (0.90–1.01)	0.073
Diastolic BP	1.10 (1.05–1.15)	< 0.001	1.05 (1.00–1.10)	0.033	1.04 (0.99–1.09)	0.087
Height	0.81 (0.78–0.85)	< 0.001	0.95 (0.90–1.01)	0.092	1.00 (0.95–1.06)	0.963
Weight	1.03 (0.98–1.07)	0.263	0.94 (0.85–1.05)	0.264	1.02 (0.92–1.14)	0.697
Waist circumference	1.19 (1.14–1.25)	< 0.001	1.07 (0.97–1.18)	0.187	0.99 (0.90–1.10)	0.885
Glucose	1.05 (1.01–1.10)	0.013	0.96 (0.91–1.01)	0.109	0.95 (0.90–1.00)	0.037
Total cholesterol	1.02 (0.98–1.07)	0.378	0.98 (0.93–1.02)	0.313	0.98 (0.94–1.03)	0.494
HDL-C	0.83 (0.79–0.87)	< 0.001	0.90 (0.85–0.94)	< 0.001	0.94 (0.89–0.99)	0.016
Triglycerides	1.16 (1.11–1.20)	< 0.001	1.10 (1.05–1.14)	< 0.001	1.07 (1.02–1.11)	0.003
AST	1.11 (1.06–1.15)	< 0.001	1.05 (1.01–1.09)	0.007	1.04 (1.00–1.08)	0.041
ALT	1.10 (1.06–1.14)	< 0.001	1.05 (1.01–1.10)	0.014	1.03 (0.99–1.08)	0.145
Hemoglobin	1.02 (0.97–1.07)	0.466	1.00 (0.95–1.05)	0.966	0.99 (0.94–1.04)	0.615
Hematocrit	0.92 (0.88–0.96)	< 0.001	0.91 (0.87–0.95)	< 0.001	0.91 (0.87–0.95)	< 0.001
BUN	1.07 (1.02–1.11)	0.004	0.92 (0.88–0.97)	0.002	0.93 (0.88–0.98)	0.004
Creatinine	1.08 (1.04–1.11)	< 0.001	1.05 (1.02–1.09)	0.002	1.05 (1.01–1.08)	0.007
WBC	1.04 (0.99–1.08)	0.133	1.03 (0.98–1.08)	0.246	0.99 (0.94–1.03)	0.575
RBC	0.89 (0.85–0.94)	< 0.001	0.92 (0.88–0.96)	< 0.001	0.92 (0.88–0.96)	0.001

*HDL-C* high-density lipoprotein cholesterol, *AST* aspartate aminotransferase, *ALT* alanine aminotransferase, *BUN* blood urea nitrogen, *WBC* white blood cell, *RBC* red blood cell.

Crude Model: unadjusted model, Model 1: adjusted for age and BMI, Model 2: adjusted for age, BMI, income, education, occupation, number of household members, marital status, stress, smoking, alcohol consumption, menstruation, and pregnancy.

Regarding biochemical indices, the depression group was more likely to have higher glucose levels than the control group in the crude model and model 2. Subjects with depression tended to have lower HDL-C levels than individuals in the control group in all models. Additionally, in all models, individuals in the depression group were significantly more likely to have higher triglyceride levels, higher AST levels, lower hematocrit levels, higher BUN levels, higher creatinine levels, and lower RBC levels than individuals in the control group. ALT levels were associated with depression in the crude model and model 1, but this association disappeared in model 2.

## Discussion

In this large-scale cross-sectional study regarding anthropometric indices related to obesity, our results suggested that men in the depressive disorder group were significantly more likely to have a lower height and weight than men in the control group, but this was not true for women. Abdominal obesity (waist circumference) was not associated with depression in either men or women. BMI was associated with the disorder in women but not in men in the crude model. Regarding biochemical indices, our results showed that compared to men in the control group, men in the depression group were significantly more likely to have higher triglyceride levels and tended to have lower hematocrit and BUN levels. Women in the depression group tended to have higher triglyceride, AST, BUN, and creatinine levels and lower HDL-C, hematocrit, and RBC counts than those in the control group.

To date, many studies have been performed to reveal association between biochemical indices and depression disorder, but the associations remain unclear because the results of previous studies are contradictory: significant associations were found with triglyceride level^31,32,35^ as well as no association^33,41^, associations were found with total cholesterol level^41^ as well as no association^31,33^, associations were found with HDL-C level^33–36^ as well as no association^31,41^, associations were found with WBC^37^ as well as no association^37,41^, associations were found with RBC^29,37,38^ as well as no association^41^, associations were found with hemoglobin levels in both genders^38^ or only in men^37^ and no association was found as well^41^, associations were found with hematocrit level^38^ as well as no association^37,41^, associations were found with creatinine level^33^ as well as no association^41^, and significant associations were found with glucose level^33,43^ as well as no association^29,41^. For example, Tyrovolas et al*.*^32^ reported that hypercholesterolemia was associated with depressive symptoms because hypercholesterolemic subjects had higher depression levels than normal subjects. Hamidifard et al.^41^ assessed plasma levels of lipoprotein between healthy subjects and subjects with major depression in Iranian adults and reported that subjects with major depression were more likely to have decreased levels of total cholesterol and LDL-C. Additionally, they argued that triglycerides, creatinine, glucose, HDL-C, WBC, RBC, hemoglobin, hematocrit, and BMI were not associated with depressive disorder. A study by Vandoolaeghe et al.^38^ tested the differences in hematological indices between healthy and major depression subjects and the effects before and after treatment with antidepressants. They found that participants with major depression had lower levels of RBCs, hematocrit, and hemoglobin than healthy participants, and after treatment with antidepressive drugs for 5 weeks, there was no effect on the biochemical indices. Peng et al.^33^ mentioned that major depression patients were more likely to have lower ALT, BUN, and creatinine levels and to have higher HDL-C and glucose levels than normal control subjects in Chinese adult men and women. They argued that depression was not associated with total cholesterol or triglyceride levels. Another study by Peng et al.^43^ revealed that fasting blood glucose concentration was associated with major depression in the Chinese population, irrespective of age, sex, and other potential confounders. A study by Van Reedt Dortland et al.^44^ reported that patients with current depression had higher triglyceride levels and lower HDL-C than patients with remitted depression and control subjects in Dutch adults, but these associations disappeared after adjustment for alcohol consumption, smoking, BMI, and education level. Another study by Lehto et al.^36^ suggested that subjects with long-term depression tended to have lower HDL-C levels than healthy subjects. In a meta-analysis, Wei et al.^35^ documented that subjects with first-episode depression were more likely to have higher triglycerides and lower HDL-C than healthy subjects. Our findings were consistent with the results of previous studies, suggesting an association with triglycerides^31,32,35^, hematocrit^38^, BUN^33^, and glucose^33,43^. Additionally, our findings were similar to the results of previous studies indicating no associations with WBCs^37,41^ or with hemoglobin^41^. In contrast to the association with HDL-C^33–36^ and the association with creatinine^33^ in previous studies, our findings showed these associations in only women but not men. Our results disagreed with previous results that RBCs were not associated with depression^41^ or were associated with depression in only men^37^ because our findings showed that the association between RBCs and depression was stronger in women than men. Furthermore, our findings suggested that depressive disorder was related to AST in only women but not in men and was not related to total cholesterol in women. We postulate that one of the reasons for the difference in association is that these associations or magnitudes of associations may differ according to age group, ethnic group, country, and sex^45,46^.

The association between anthropometric indices and depression is still under debate. Some studies argued that anthropometric indices were linked to depression disorder. For example, Herva et al.^21^ argued that in males, abdominal obesity may be closely associated with depression, and in adolescent and adult females, overweight and obesity may be a risk factor for depression in Caucasians based on follow-up studies of individuals aged 14 to 31 years in a longitudinal Northern Finland 1966 Birth Cohort Study. Bjerkeset et al.^47^ found an inverse association between depression and adult height in a model adjusted for age and sex, but the association disappeared in a model adjusted for more confounders in Norway. Additionally, they argued that BMI was positively associated with depression. Singh et al.^28^ reported that depression may be a factor in weight gain, but weight gain or loss may not be a cause of depression in Australian women. Boutelle et al.^22^ suggested that obesity was associated with future depressive symptoms in adolescent females, even though obesity was not related to major or clinical depression. Blasco et al.^23^ found an interconnection between depression and obesity. They mentioned that depression increased the risk for obesity in African American adolescent males, and obesity increased the risk for depression in females based on a systematic review study. Lasserre et al.^6^ reported that the atypical subtype of major depressive disorder was a powerful risk factor for obesity in accordance with an increase in BMI and waist circumference in both sexes based on a prospective population-based cohort study in Switzerland. Luppino et al.^24^ argued that obesity was associated with a higher risk of depression in Americans than in Europeans, and overweight was associated with an increased risk of depression in adults but not young subjects according to a systematic review and meta-analysis. Godin et al.^25^ documented that compared with normal BMI, high BMI was a risk factor for developing depression in elderly subjects in France. A study conducted by Sachs-Ericsson et al.^45^ suggested that BMI was a predictor of depressive symptoms, and the effect of BMI was stronger in African Americans than in whites among elderly adults, irrespective of sex. Ma and Xiao^26^ reported that BMI was related to the probability of depressive symptoms and major depression, and greater waist circumference was significantly associated with major depression and depressive symptoms, independent of BMI. A study by Williams et al.^27^ revealed that female subjects with a lifetime history of depressive disorder tended to have greater weight, BMI, waist circumference, and body fat than those without a lifetime history of depression in Australia. Anderson et al.^46^ examined the association between depressive disorder and weight change in a prospective longitudinal study conducted from childhood to adulthood in the US and revealed that depressive disorder was associated with high BMI in women but not men. On the other hand, several studies argued that depression was not associated with obesity indices. Von Zimmermann et al.^42^ revealed that BMI, weight, height, and waist circumference were not different between the major depressive episode group and the healthy control group in German men and women. A study by Wu et al.^31^ suggested that BMI and waist-to-hip ratio were not associated with depressive symptoms in Chinese adults. Hach et al.^40^ found that waist circumference in adult women was not associated with depressive disorder in Germany. Two studies by Ormonde Do Carmo et al.^29^ and Hamidifard et al.^41^ reported that BMI was not associated with depressive disorder. Our findings were consistent with the results of previous studies indicating that depression was associated with weight^28^ and was not associated with waist circumference^40,42^. Additionally, our crude model results were linked to those of previous studies suggesting that depression was related to BMI in only women^23,46^.

The present study had some limitations. This study population was limited to the Korean population despite the large sample size, and our results cannot be guaranteed to be similar to findings in other races and countries because of differences in sociodemographic characteristics, economic statuses, and environmental statuses of countries. Additionally, our findings cannot indicate causal relationships due to the cross-sectional nature of the study. The depression diagnosis of the subjects was determined via face-to face interviews with well-trained staff. Therefore, diagnostic information was limited by the subjects’ answers. Additionally, our results had statistical limitations because this study did not consider the statistical significance of a large sample size. Even though our results had these limitations, the statistical results and findings in the present study are powerful because of the large-scale data from a nationally representative sample of the Korean population (KNHANES) from 2007 to 2018.

## Methods

### Subjects and data source

This was a large-scale cross-sectional study. This study was designed to identify risk factors for depressive disorder among biochemical and anthropometric indices in South Korea. The data used in this study were obtained from the Korea National Health and Nutrition Examination Survey (KNHANES) from 2007 to 2018, which was conducted by Korea Centers for Disease Control and Prevention (KCDC). The KNHANES IV-VII was approved by the Institutional Review Board of the KCDC (2018-01-03-P-A) and conducted in accordance with the Declaration of Helsinki. All participants in this survey signed an informed consent form. Additionally, we obtained ethics approval from the Institutional Review Board of the Korea Institute of Oriental Medicine (KIOM) for the use of the KNHANES data (IRB No. I-2007/006-003).

**Figure 1:**
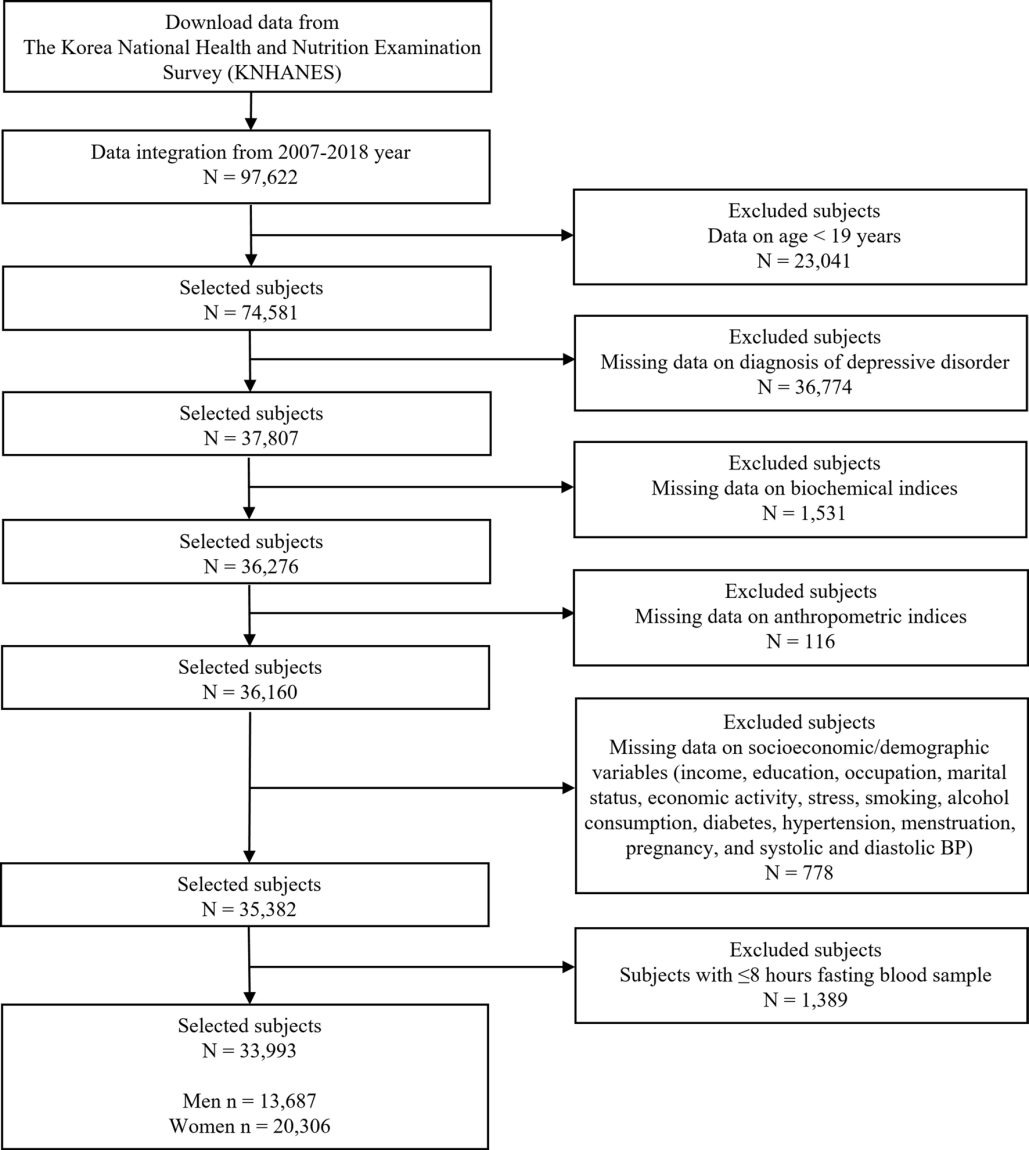
Sample selection procedure.

We collected data from KNHANES IV to KNHANES VII (2007–2018). Initially, the data included 97,622 subjects, and we included 33,993 subjects in our analyses according to inclusion and exclusion criteria: (1) included only adult subjects (aged 20–80 years), (2) excluded subjects without information of diagnosis of depression disorder by a doctor, (3) excluded subjects without anthropometric and biochemical indices, (4) excluded subjects without major socioeconomic/demographic data such as occupation, education, smoking, alcohol consumption, BP, and chronic disease diagnosis in both men and women and menstruation and pregnancy in women, and (5) excluded subjects with ≤ 8 h fasting blood sample. Details on sample selection procedure are shown in Fig. 1.

### Definitions

The KNHANES aims to obtain representative and reliable statistics of the health and nutrition information of the Korean population. For the identification of subjects with depressive disorder, the question “Do you have depressive disorder diagnosed by a physician?” was asked in face-to face interviews with well-trained staff during the heath interviews and examinations conducted during the KNHANES IV–VII surveys; all subjects answered “Yes”, “No, or “Not applicable” according to the KCDC guidelines. Therefore, the depressive disorder group included subjects who answered “Yes”, and the control group consisted of subjects who answered “No” or “Not applicable”. Additionally, interviews were conducted to obtain socioeconomic/demographic characteristics. For example, personal income consisted of quintiles: low (coded as 1), lower-middle (2), middle (3), upper-middle (4), and high (5). Education level was divided into four groups: completion of elementary school or less (1), middle school (2), high school (3), and university or higher (4). Smoking was divided into two groups: nonsmoking (0) and smoking (1) (current smoking and more than 100 cigarettes in a lifetime). Regarding diseases, diabetes was identified by answers of “No” (0), “Yes” (1), and “Not applicable” (1) to the question “Have you ever been diagnosed with diabetes by a doctor?”.

### Measurements

Anthropometric and biochemical indices were tested according to standardized protocols by trained technicians or medical personnel. Height was measured to the nearest 0.1 cm (Seca 225, Seca, Germany), and weight was measured to the nearest 0.1 kg (GL-6000-20, G-tech, Korea). BMI was calculated as weight (in kilograms) divided by height (in meters squared). BP was measured three times using a standard mercury sphygmomanometer (Baumanometer; WA Baum Co., Copiague, NY, USA) and defined as the mean value of the second and third measurements. Blood tests were analyzed after ≥ 8 h fasting. Total cholesterol, triglyceride, HDL-C, AST, ALT, and glucose levels were analyzed by an ADVIA 1650 (Siemens, New York, USA) and Hitachi Automatic Analyzer 7600 (Hitachi, Tokyo, Japan), and WBC, RBC, hemoglobin, and hematocrit were analyzed by an ADVIA 120 (Siemens, New York, USA) and XE-2100D and XN-9000 (Sysmex, Kobe, Japan). All equipment used in the KNHANES survey was calibrated periodically. Socioeconomic/demographic characteristics (income, education, occupation, marital status, economic activity, stress, smoking, alcohol consumption, menstruation, pregnancy, diabetes and hypertension diagnosis) were collected via a face-to face interview and a self-administered questionnaire.

### Statistical analysis

All statistical analyses were performed using SPSS Statistics 23 for Windows (SPSS, Inc., Chicago, IL, US). Continuous and categorical variables are summarized as the mean (± standard deviation) and the frequency (percentage). The chi-square test was used to compare categorical variables between the control and depressive disorder groups. Binary logistic regression was used to examine the association between the control and depressive disorder groups after standardization transformation (mean = 0 and standard deviation = 1). The crude model included only one explanatory variable. For the adjustment of potential confounders, we considered adjusted models. Model 1 included one explanatory variable and covariates (age and BMI). Model 2 included one variable and the covariates of model 1, plus income, education, occupation, number of household members, marital status, stress, smoking, and alcohol consumption in men and income, education, occupation, number of household members, marital status, stress, smoking, alcohol consumption, menstruation, and pregnancy in women. To select the covariates for adjustment, we referred to the sociodemographic characteristics listed in previous studies^5,14–28^. Thus, sociodemographic variables related to depressive disorder were used covariates. Odds ratios are presented with 95% confidence intervals (CIs), and a p-value < 0.05 was considered significant.

## Data Availability

Data used in this study are available from KNHANES (KCDC). Anyone can freely access the data (https://knhanes.cdc.go.kr/knhanes/main.do and http://www.kdca.go.kr/).
